# TurboID-based mapping of organelle membrane protein interactomes with digitonin-permeabilization

**DOI:** 10.52601/bpr.2025.240051

**Published:** 2025-08-31

**Authors:** Yang Sun, Lan Yang, Jingzi Zhang, Pu Tian, Shue Chen, Lei Fang, Zhi Hong

**Affiliations:** 1 Centre for Cellular Biology and Signaling, Zhejiang University-University of Edinburgh Institute, Haining 314400, Zhejiang, China; 2 University of Edinburgh Medical School, Biomedical Sciences, College of Medicine & Veterinary Medicine, University of Edinburgh, Edinburgh, EH8 9JZ, United Kingdom; 3 State Key Laboratory of Pharmaceutical Biotechnology, Chemistry and Biomedicine Innovation Center, Medical School of Nanjing University, Nanjing 210000, China; 4 International Center for Aging and Cancer, Hainan Medical University, Haikou 571199, China

**Keywords:** Digitonin-permeabilization, Organelle interactomes, Proximity labeling, Protein–protein interactions, REEP1/REEP6

## Abstract

Protein–protein interactions at organelle membranes bridge organelles in close proximity, facilitating regulated metabolite exchange and maintaining cellular homeostasis. Enzyme-catalyzed proximity labeling (PL) has been widely used to uncover the molecular composition of these interactions, but excessive labeling of irrelevant cytosolic proteins complicates data analysis. To address this, we developed a streamlined protocol that combines the TurboID system with digitonin-permeabilization to efficiently map protein interactions at organelle membranes in live mammalian cells. Digitonin selectively permeabilizes the plasma membrane, removing cytosolic proteins while preserving the integrity of inner membranes like the ER and mitochondria. This approach enhances spatial resolution in proteo-mic analysis, enabling a more precise map for protein interactome. Using this method, we successfully achieved proximal labeling of ER-localized proteins REEP1 and REEP6 to decipher their interaction networks, demonstrating its applicability for studying membrane-associated interactions with greater clarity and reduced contamination.

## INTRODUCTION

Intracellular compartmentalization enables organelles to conduct biochemical processes in a spatiotemporally regulated manner, yet it also poses challenges in coordinating metabolic fluxes across different organelles (Loewen *et al*. 2003; Xue *et al*. 2017). The organelle interactome refers to the intricate network of interactions between organelles, including protein–protein interactions, signal transduction, metabolite exchange, and other functional couplings (Valm *et al.* 2017). Protein complexes on organelle membranes anchor adjacent organelles, forming membrane contact sites (MCSs), which are essential for mediating inter-organelle communication and functional coordination (Csordás *et al.* 2006; Prinz *et al.* 2020). Therefore, developing large-scale methods to unravel protein–protein interaction networks among organelles in living cells is of great importance (Huang *et al.* 2020).

Traditional techniques such as subcellular fractionation, yeast two-hybrid (Y2H), and affinity purification (AP), have greatly advanced our understanding of organelle interactomes. However, these methods faced limitations in detecting weak and transient interactions, especially those at MCSs (Aniento and Gruenberg, 2003; Brückner *et al.* 2009; Dunham *et al.* 2012). To overcome these challenges, enzyme-catalyzed proximity labeling (PL) has emerged as a powerful alternative, enabling the tagging of endogenous interaction partners of specific protein “baits” in living cells. PL enzymes, such as biotin ligases (*e.g*., BioID) and peroxidases (*e.g*., APEX), label proteins within a 1–10 nm range. The biotinylated proteins are then captured using streptavidin-coated beads and identified by mass spectrometry (MS) (Branon *et al.* 2018; Lobingier *et al.* 2017; Roux *et al.* 2012).

Among the most widely employed proximity labeling techniques are BioID, TurboID, and APEX, each offering distinct strengths and limitations. BioID utilizes a biotin ligase and has been extensively used to map protein interaction networks in living cells. However, its relatively slow labeling kinetics, requiring 18–24 hours has limited its utility for capturing transient and rapidly fluctuating interactions (Roux *et al.* 2012). TurboID, an engineered variant of BioID, overcomes this limitation by significantly accelerating the biotinylation process, enabling efficient labeling within minutes (Branon *et al.* 2018; Cho *et al.* 2020). This rapid labeling ability makes TurboID particularly well-suited for studying dynamic and short-lived interactions. APEX utilizes peroxidase to achieve proximity labeling with faster labeling kinetics and high spatial resolution. However, its reliance on hydrogen peroxide (H_2_O_2_) as a cofactor introduces oxidative stress, disrupting cellular physiology and restricting its application in H_2_O_2_-sensitive systems (Hung *et al.* 2014; Lam *et al.* 2015).

In this study, TurboID was chosen for its rapid labeling kinetics, low toxicity, and minimal perturbation to cellular processes. The efficiency in capturing transient interactions makes it particularly advantageous for the study of dynamic interactions among membrane proteins. In addition, TurboID requires only biotin as an exogenous reagent, thereby minimizing the risk of experimental artifacts and ensuring greater compatibility with diverse experimental conditions. These features make TurboID ideal for studying protein interactions in subcellular locations. However, excessive labeling of cytosolic proteins can complicate data analysis, particularly when investigating membrane-associated protein interactions on organelles, creating challenges for subsequent functional studies.

To address cytosolic contamination, previous studies have primarily employed two strategies: (1) the Ratiometric SILAC approach (Hung *et al.* 2017); (2) subcellular fractionation or organelle isolation (Bersuker *et al.* 2018). While effective, these methods are labor-intensive and costly. In response, we developed a simpler technique that retains the same spatial resolution for proteomics. Digitonin, a steroidal glycoside, selectively permeabilizes cholesterol-rich membranes such as the plasma membrane (PM) while preserving cholesterol-poor membranes like those of the endoplasmic reticulum (ER) and mitochondria (Foucher *et al.* 2006; Plutner *et al.* 1992). This selective permeabilization enables the removal of cytosolic components while keeping organelle structures intact.

Our protocol combines TurboID-based proximity labeling with digitonin treatment to eliminate cytosolic contaminants. Biotinylated proteins are then analyzed by mass spectrometry to identify interaction partners. This method reduces cytosolic protein interference and enhances the precision of protein–protein interaction mapping on organelle membranes. Using this approach, we successfully mapped the interaction networks of the ER membrane-shaping proteins REEP1 and REEP6.

## STEP-BY-STEP PROCEDURE

### Step 1: Cloning of TurboID fusion constructs [TIMING 2–3 days]

#### Step 1.1: Preparation of inserts and vectors

Amplify the TurboID fragment from the 3× HA-TurboID-NLS construct, the Myc-REEP1 and Myc-REEP6 fragments from the pCMV-Myc-REEP1 and pCMV-Myc-REEP6 constructs, respectively, using Phanta Max Super-Fidelity DNA Polymerase. Add the following reagents to a PCR tube: Template DNA 100 ng, Phanta Max Super-Fidelity DNA Polymerase 1 μL, 1× PCR Buffer 25 μL, dNTP Mix (10 mmol/L) 1 μL, Forward Primer (10 μmol/L) 2 μL, Reverse Primer (10 μmol/L) 2 μL, ddH_2_O: up to 50 μL.

Program the PCR thermocycler with the following settings: (1) Initial denaturation: 95°C for 3 min; (2) Denaturation: 95°C for 15 s; (3) Annealing: calculate based on primer Tm, 30 s; (4) Extension: 72°C for *X* s (depends on fragment size, typically 1 kb/min); (5) Final extension: 72°C for 5 min; (6) Total cycles: 30–35 cycles, depending on template concentration and complexity.

Step 1.1.1: Digest the plvx-IRES-puro vector with EcoRI and BamHI at 37°C for 3 h.

#### Step 1.2: Agarose gel electrophoresis

Step 1.2.1: Prepare a 1% (*w*/*v*) agarose gel by dissolving 1 g of agarose in 100 mL of TAE buffer. Heat until boiling, cool to approximately 60°C, and add 5 μL DNA green stain before allowing it to set.

Step 1.2.2: Run the PCR products and digested vector on a 1% agarose gel at 120 V for 35 min.

Step 1.2.3: Excise gel bands under UV light, dissolve in GDP buffer, centrifuge, wash with GW buffer containing 80% ethanol, and elute DNA with pre-warmed ddH_2_O.

#### Step 1.3: Recombination and transformation

Step 1.3.1: Mix the DNA fragments at a 1:1:1 molar ratio with the recombinant enzyme. Incubate the mixture at 37°C for 30 min, then place it on ice to stop the reaction.

Step 1.3.2: Thaw 30 μL of competent Stbl3 *E. coli* cells on ice for 10 min. Mix the cells with the ligation product on ice for 20 min.

Step 1.3.3: Heat shock briefly at 42°C for 45 s, then cool on ice for 2 min.

Step 1.3.4: Add 1 mL of blank LB medium and incubate at 37°C with shaking at 220 r/min for 1 h.

Step 1.3.5: Spread the transformed cells onto LB agar plates containing 100 µg/mL ampicillin and incubate overnight at 37°C.

Step 1.3.6: Pick colonies and inoculate into 5 mL LB medium with 100 µg/mL ampicillin. Incubate overnight at 37°C with shaking at 220 r/min.

Step 1.3.7: Isolate the plasmids and confirm the presence of the correct insert by sequencing.

### Step 2: Generating stable cell lines [TIMING 10–14 days]

#### Step 2.1:Lentivirus production

Step 2.1.1: Seed HEK293T cells in 6-well plates and incubate overnight.

Step 2.1.2: Transfect cells at 60% confluency using polyethyleneimine (PEI) 25K. Use 1.5 μg of psPAX2, 0.5 μg of pMD2.G, and 2 μg of plvx-Myc-REEP1/6-TurboID for each well.

**[TIP]** Use PEI 25K to plasmid at a ratio of 2:1 for optimal transfection efficiency.

Step 2.1.3: After 6 h, replace the culture medium.

Step 2.1.4: Harvest the supernatant at 48 and 60 h post-transfection, centrifuge, and filter through a 0.45-μm filter. Store the viral supernatant at 4°C.

**[TIP]** For long-term storage, aliquot the virus and keep it at −80°C. Avoid repeated freeze-thaw cycles to maintain its viability.

#### Step 2.2: Transduction

Step 2.2.1: Seed the HEK293T and U2OS cells in 12-well plates and incubate overnight.

Step 2.2.2: Mix 0.67 mL lentivirus with 1.34 mL of complete culture medium (1:3 ratio). Add 1.6 µL of 10 mg/mL polybrene.

**[Critical step]** Optimize the virus-to-medium ratio to enhance transduction efficiency and reduce cell death during antibiotic selection.

Step 2.2.3: Incubate cells with the virus mixture for 6 h, then refresh the medium with 1 mL of complete medium.

#### Step 2.3: Selection of transduced cells

Step 2.3.1: After 24 h of transduction, add puromycin at a working concentration of 1 μg/mL to select cells expressing Myc-REEP-turboID.

Step 2.3.2: Continue selection with puromycin for 4–5 days.

**[TIP]** Regularly change the medium and monitor cell growth. Ensure complete cell death in the untransduced control group.

Step 2.3.3: Transfer selected cells to puromycin-free medium and check for mycoplasma contamination.

NOTE: Disinfect all materials that have been in contact with lentivirus for at least 48 h before disposal.

### Step 3: Validation of stable cell lines [TIMING 3–4 days]

#### Step 3.1: Validation by western blots

Step 3.1.1: Harvest Myc-REEP1/6-TurboID HEK293T cells at 90% confluency in a 12-well plate.

Step 3.1.2: Discard the medium, and wash cells with 1× cold PBS on ice.

Step 3.1.3: Add 75 µL of lysis buffer to both the wide-type and stable cell lines.

Step 3.1.4: Collect the cell pellets, rotate at 4°C for 40 min, and centrifuge at 12,000 r/min for 20 min.

Step 3.1.5: Collect the supernatant and mix it with 25 µL of 4× protein loading buffer, boiling at 100°C for 15 min.

Step 3.1.6: Load 20 µL of samples onto a 10% SDS-PAGE gel, starting electrophoresis at 80 V for 20 min, then increasing to 110 V for 70 min.

Step 3.1.7: After electrophoresis, assemble the polyacrylamide gel, nitrocellulose membranes, and transfer clips. Transfer the membranes at 350 mA for 100 min.

Step 3.1.8: Submerge the transferred membrane in 40 mL of 10% skimmed milk and shake at 40 r/min for 1 h.

Step 3.1.9: Transfer the membrane to 40 mL of 1× TBST and shake at 70 r/min for 2 min.

Step 3.1.10: Incubate the membrane in primary antibody solutions (HRP-Conjugated Streptavidin, 1:10,000 diluted with primary antibody dilution buffer) for 1 h at room temperature or overnight at 4°C.

Step 3.1.11: Wash the membrane three times with 15 mL of 1× TBST for 15 min each.

Step 3.1.12: Incubate with ECL substrate for 1 min and capture images using a chemiluminescent imaging system.

Step 3.1.13: Repeat the incubation of the membrane with anti-Myc antibody for 2 h at room temperature or overnight at 4°C.

Step 3.1.14: Wash the membranes three times with 15 mL of 1× TBST. Incubate the membrane in a secondary antibody solution (anti-rabbit 1:10,000 diluted in 1× TBST).

Step 3.1.15: Wash the membrane three times with 15 mL of 1× TBST for 15 min each.

Step 3.1.16: Western blot data analysis.

#### Step 3.2: Validation by microscopy

Step 3.2.1: Seed Myc-REEP1/6-turboID U2OS cells on coverslips in a 24-well plate at 60% confluency and incubate overnight.

Step 3.2.2: Transfect the cells with Sec61β-GFP using Lipofectamine 2000. For each well, prepare solution A by diluting 200 ng Sec61β-GFP in 25 μL Opti-MEM and solution B by diluting 0.4 μL Lipofectamine 2000 in 25 μL Opti-MEM.

Step 3.2.3: Gently mix Solutions A and B at room temperature for 5 min. After that, combine Solution A with Solution B and let stand for an additional 20 min.

Step 3.2.4: Add the transfection mixture to the cells and gently mix.

Step 3.2.5: After 5 h, replace the medium with 500 μL of complete culture medium. Incubate cells for another 15–18 h.

Step 3.2.6: Fix cells with 500 µL of 4% paraformaldehyde at room temperature for 10 min, then wash three times with 500 µL of pre-warmed PBS for 15 min each.

Step 3.2.7: Block cells with 500 µL of blocking buffer (1× PBS, 0.4% Triton X-100, 1% BSA) for 30 min at room temperature.

Step 3.2.8: Incubate cells with 300 µL of primary antibody (Rabbit anti-Myc, 1:400) in blocking buffer for 2 h at room temperature or overnight at 4°C. Wash three times with 1× PBS.

**[TIP]** Ensure the coverslip is fully submerged in the antibody solution.

Step 3.2.9: Incubate cells in the dark with the secondary antibody (anti-Rabbit Alexa Fluor 555, 1:1000) for 1 h and wash three times with 1× PBS.

Step 3.2.10: Stain cells with DAPI solution (0.33 µg/mL) and wash three times with PBS and once with ddH_2_O.

Step 3.2.11: Mount the coverslips with mounting medium and store at 4°C.

Step 3.2.12: Acquire images using a Zeiss LSM 880 laser confocal microscope with a 63× oil objective (NA 1.4).

#### Step 3.3: Validation by TurboID activity

Step 3.3.1: Seed Myc-REEP1/6-turboID HEK293T cells in a 6-cm plate at 90% confluency and incubate overnight.

Step 3.3.2: Incubate cells with 1 mL of 500 μmol/L biotin at 37°C, 5% CO_2_ for 10 min. For control groups, add 0.5% DMSO.

**[TIP]** Ensure the biotin solution covers the cells evenly.

Step 3.3.3: To halt labeling, place the dish on ice, and discard the medium completely.

Step 3.3.4: Wash cells five times with 3 mL of ice-cold 1× PBS.

**[TIP]** Keep the process on ice to maintain integrity.

Step 3.3.5: Lyse cells with 300 μL of RIPA lysis buffer on ice for 10 min.

Step 3.3.6: Scrape cells and transfer them to a centrifuge tube, centrifuge at 10,200 r/min for 10 min at 4°C.

Step 3.3.7: Transfer the supernatant into a new tube.

Step 3.3.8: Take 10 μL for protein quantification, and mix 20 μL with 7 μL 4× loading buffer for the whole cell lysate. Boil at 100°C for 15 min.

Step 3.3.9: Incubate the remaining supernatant with 20 μL of pre-washed streptavidin-coated beads for 1 h at room temperature with rotation. Then move to 4°C and rotate overnight.

Step 3.3.10: Wash the beads sequentially with 1 mL RIPA lysis buffer twice, 1 mL 1 mol/L KCl once, 1 mL 0.1 mol/L Na_2_CO_3_ once, 1 mL 2 mol/L urea in 10 mmol/L Tris-HCl (pH 8.0) once, and finally with 1 mL RIPA lysis buffer twice.

Step 3.3.11: Boil the beads in 20 μL of 4× loading buffer at 100°C for 15 min and analyze by western blots.

### Step 4: Isolation and enrichment of biotinylated membrane proteins for Mass spectrometry analysis [TIMING 3–4 days]

#### Step 4.1: Biotin labeling

Step 4.1.1: Coat a 10-cm dish with 4 mL of 5 μg/mL poly-L-lysine and incubate overnight at 4°C. Considering the rigorous washing steps (up to ten times) performed before cell lysis, poly-L-lysine is used to enhance cell adhesion of HEK293T cells for maintaining sample integrity throughout the experimental process.

Step 4.1.2: Seed Myc-REEP1/6-turboID HEK293T cells in the dish at 1.3 × 10^7^ cells and culture for 14 h.

Step 4.1.3: Incubate cells with 3 mL of 500 μmol/L biotin at 37°C, 5% CO_2_ for 10 min. For control groups, add 0.5% DMSO.

Step 4.1.4: Place the dish on ice to stop labeling, discard the medium, and wash cells on the plate five times with 3 mL ice-cold 1× PBS.

#### Step 4.2: Digitonin permeabilization

Step 4.2.1: Gently add 3 mL of cold digitonin solution or blank solution to the cells.

**[TIP]** Prepare the digitonin buffer fresh on the day of the experiment.

Step 4.2.2: Incubate cells at 4°C with shaking at 100 r/min for 10 min.

Step 4.2.3: Discard the supernatant and wash cells on the plate five times with ice-cold 1× PBS.

**[Critical step]** Ensure PBS is completely removed.

**[TIP]** Collect the supernatant as the “cytosolic fraction” sample. Verify by western blots to confirm successful cytosolic extraction without membrane protein contamination.

Step 4.2.4: Lyse cells with 700 μL of RIPA buffer on ice for 10 min.

Step 4.2.5: Collect the lysate and centrifuge at 10,200 r/min for 10 min at 4°C.

Step 4.2.6: Collect 20 μL of the supernatant, mix with 7 μL 4× loading buffer, and boil at 100°C for 15 min as the pre-enriched group.

#### Step 4.3: Enrichment of biotinylated proteins

Step 4.3.1: Wash 40 μL of streptavidin-coated beads three times with RIPA buffer. Incubate the beads with clarified lysate for 1 h at room temperature with rotation, then move to 4°C and rotate overnight.

Step 4.3.2: Wash the beads with 1 mL RIPA lysis buffer twice, 1 mL 1 mol/L KCl once, 1 mL 0.1 mol/L Na_2_CO_3_ once, 1 mL 2 mol/L urea in 10 mmol/L Tris-HCl (pH 8.0) once, and finally with 1 mL RIPA lysis buffer twice.

Step 4.3.3: Boil 5 μL of beads in 4× loading buffer for 15 min at 100°C and analyzed by western blots.

Step 4.3.4: Wash the remaining beads five times with 1 mL 0.5 mol/L Triethylammonium bicarbonate (TEAB).

### Step 5: Mass spectrometry analysis [TIMING 7 days]

#### Step 5.1: Protein digestion and labeling

Step 5.1.1: Dilute the streptavidin beads containing 20 μg enriched protein to 100 μL with 0.5 mol/L TEAB buffer and reduce the protein with 5 mmol/L Tris(2-carboxyethyl)phosphine (TCEP) at 55°C for 1 h.

Step 5.1.2: Alkylate the reduced protein with 6.25 mmol/L S-Methyl methanethiosulfonate (MMTS) for 30 min at room temperature in the dark.

Step 5.1.3: Add trypsin to the filter at a 1:50 trypsin-to-protein mass ratio for the first overnight digestion, followed by a second digestion at a 1:100 mass ratio for 4 h at 37°C.

Step 5.1.4: Collect the resultant peptides in the supernatant by centrifugation at 1000 r/min for 2 min, and label them using the iTRAQ Reagent-8 plex Multiplex Kit according to the manufacturer's instructions. Three biological replicates per group of iTRAQ experiments were conducted.

Step 5.1.5: Mix all labeled peptides, dry them using a SpeedVac, and fractionate them using a high-performance liquid chromatography (HPLC) system with a Durashell C18 column (5 μm, 100 Å, 4.6 × 250 mm).

Step 5.1.6: Obtain samples by pooling fractions. After desalting and SpeedVac drying, resuspend the samples in 3% (*v*/*v*) formic acid and 2% (*v*/*v*) acetonitrile for LC-MS/MS analysis.

#### Step 5.2: LC-MS/MS analysis

Step 5.2.1: Inject samples onto a nanoLC column (3C18-CL, 75 μm × 15 cm) of a NanoLC.2D system.

Step 5.2.2: Employ a 120-min gradient from 2% to 80% using mobile phase A (0.1% formic acid, 2% acetonitrile) and mobile phase B (0.1% formic acid, 98% acetonitrile) at 300 nL/min.

**[TIP]** The flow rate remains steady at 300 nL/min throughout the process.

Step 5.2.3: Increase solvent B from 2% to 30% over 85 min, then to 50% over 20 min, reaching 80% in 1 min, and hold for 4 min.

Step 5.2.4: Collect the original mass spectrometry data using TripleTOF 5600 system in a data-dependent acquisition mode. Acquire MS spectra from 350–1500 *m*/*z* for 250 ms. Select the 50 most intense precursors (charge state 2–5) for fragmentation, recording MS/MS spectra from 100–2000 *m*/*z* for 100 ms. Precursor ions were excluded from being reselected for 15 s.

#### Step 5.3: Data analysis

Step 5.3.1: Submit the original MS/MS data to ProteinPilot software Software for analysis. Conduct a database search against Homo sapiens in the UniProt database.

Step 5.3.2: Use the following parameters: instrument set to TripleTOF 5600, iTRAQ quantification, trypsin digestion, and cysteine modification with MMTS. Enable thorough ID, quantitation, bias correction, and background correction for protein quantification and normalization. Employ an automatic decoy database search to estimate the false discovery rate (FDR) using the PSPEP algorithm.

Step 5.3.3: Further process the data using Gene Ontology (GO) and Kyoto Encyclopedia of Genes and Genomes (KEGG) pathway enrichment tools.

**[?TROUBLESHOOTING]**

Troubleshooting advice can be found in Table 1.

**Table 1:** Troubleshooting guide

Step	Problem observed	Possible reason	Solutions
2.3.2	No transduced cells after puromycin selection	Low efficiency of lentiviral transduction	Quantify viral titer before target cell transduction; Optimize the dilution ratio
3.3.11	Fusion construct is undetectable by western blots	Incorrect protocol; Low expression level of the construct	Use a positive control; Optimize construct expression level
3.3.11	High background signal in omit-biotin control group	Excessive non-specific protein binding to streptavidin-coated beads	Increase the number of wash steps; Reduce the amount of streptavidin beads; Shorten incubation time
4.2.3	Cells detach during PBS washing	Poor cell adhesion	Optimize poly-L-lysine concentration and incubation time
4.3.3	GAPDH detected in digitonin-permeabilized groups	Residual cytoplasmic components	Use freshly prepared digitonin solution; Increase the number of wash steps
4.3.3	Low yield of biotinylated proteins	Insufficient samples or streptavidin beads used	Increase the number of cells; Increase the amount of streptavidin beads for protein enrichment

## ANTICIPATED RESULTS

### Characterization of Myc-REEP1/6-TurboID stable cell lines

**Figure 1:**
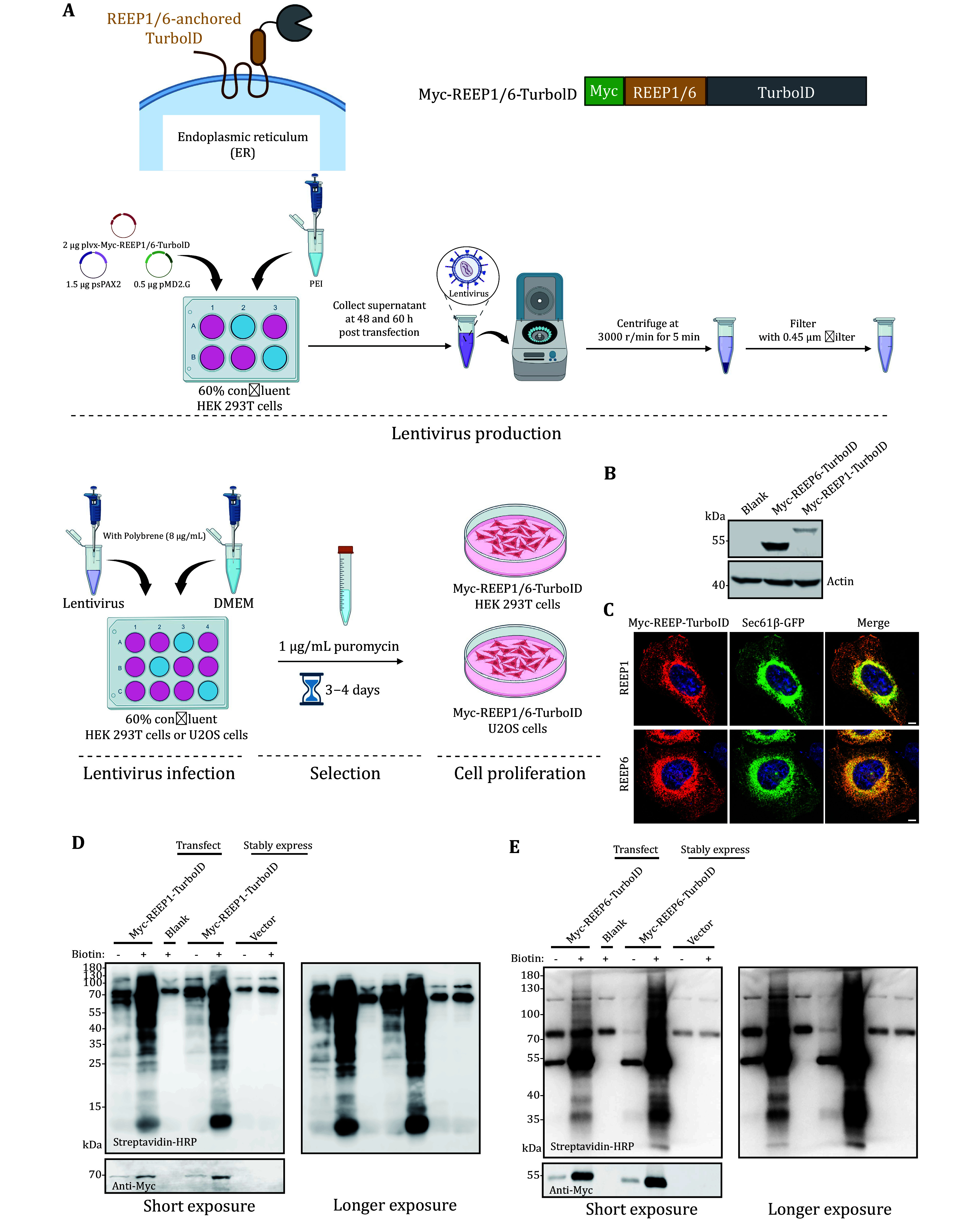
Validation of REEP-TurboID stable cell Lines by western blot and immunostaining. **A** Schematic representation of the procedure for generating stable cell lines expressing REEP-TurboID fusion proteins. Created in BioRender. **B** Western blot validation of HEK293T cells stably expressing Myc-REEP1/6-TurboID fusion constructs. REEP1 exhibited lower expression levels compared to REEP6. **C** Confocal images of U2OS cells stably expressing Myc-REEP1/6-TurboID. Sec61β-GFP was used as an ER marker. Bar: 5 μm. **D**,**E** Biotinylation activity of Myc-REEP1/6-TurbolD in HEK293T cells. Cells were treated with 500 μmol/L biotin for 10 minutes, and biotinylated proteins were analyzed by streptavidin-HRP blotting. Cells with stable expression of lentiviral expression vector (plvx-IRES-Puro) were used as a negative control

A successful proteomics mapping of REEP1 and REEP6 interactomes depends on the stable expression of TurboID fusion constructs. To achieve this, we generated stable HEK293T and U2OS cell lines expressing Myc-REEP1/6-TurboID constructs via lentiviral transduction (Fig. 1A). Western blot analysis confirmed the expression of REEP1 and REEP6 fusion proteins, with REEP1 exhibiting lower protein levels compared to REEP6. This difference in expression is likely due to the inherently lower abundance of REEP1 in HEK293T cells (Fig. 1B).

X

We validated the proper localization of the constructs using immunostaining and co-transfection with an ER-targeting marker. Fluorescence microscopy revealed that both REEP1 and REEP6 were correctly localized to the ER membrane, indicating that the TurboID tag did not alter their subcellular localization (Fig. 1C). To ensure that the TurboID fusions were functionally active, we performed a biotinylation assay. Upon biotin addition, REEP1 and REEP6 successfully biotinylated nearby proteins, confirming the ligase activity of the TurboID tag (Figs. 1D and 1E).

### Sample preparation via digitonin-permeabilization

**Figure 2:**
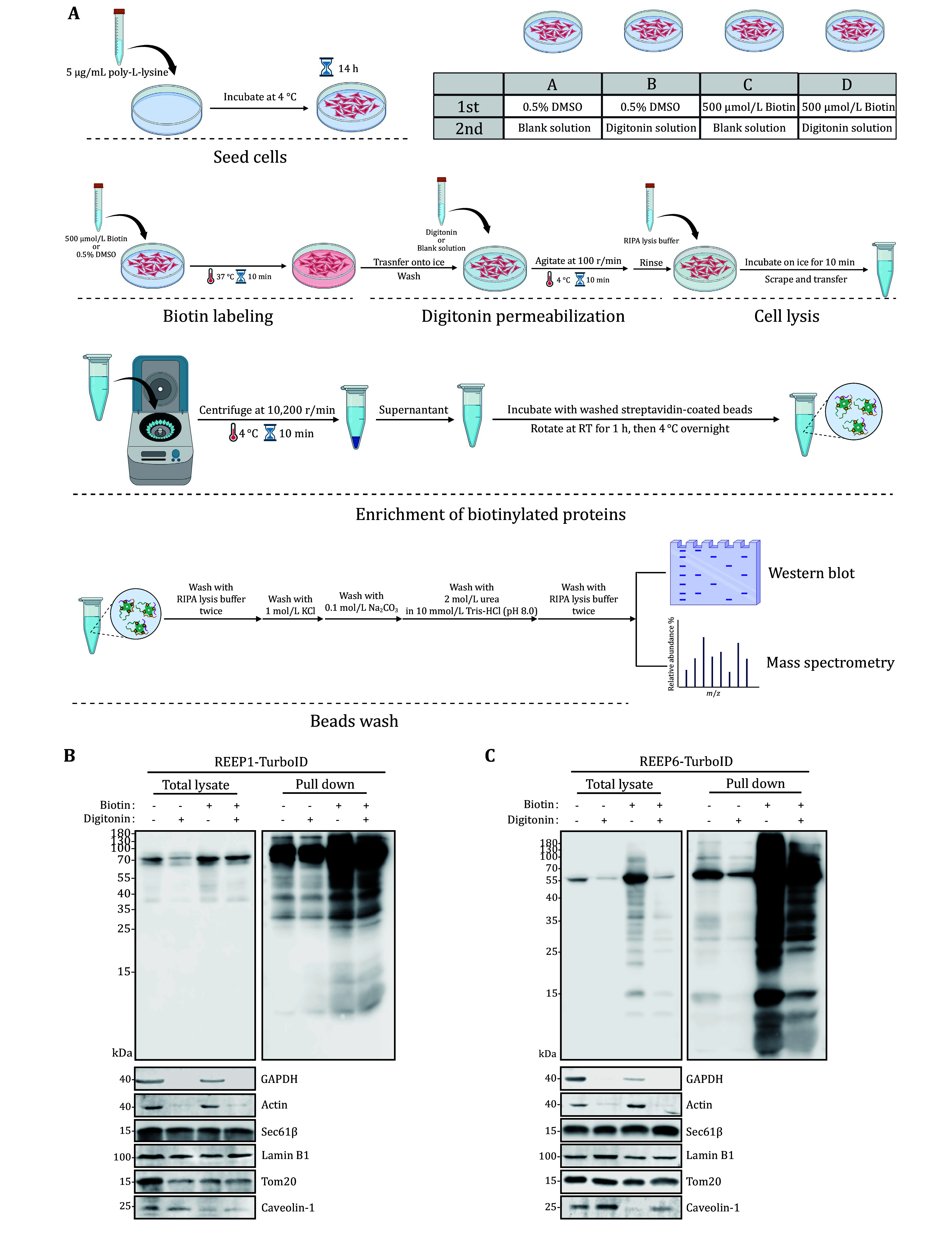
Experimental design for proteomic mapping using TurbolD and digitonin-permeabilization. **A** Schematic of the digitonin-permeabilization procedure for isolating membrane-associated biotinylated proteins. Created in BioRender. **B**,**C** Validation of digitonin-permeabilization in HEK293T by western blot. Biotinylated proteins were analyzed by streptavidin-HRP blotting. Markers for cytosolic, nuclear, plasma membrane, cytoskeleton, ER membrane, and mitochondrial membrane components were used to verify the selective retention of membrane proteins

To capture organelle-specific interactomes, digitonin permeabilization was applied following biotin labeling (Fig. 2A). HEK293T cells expressing Myc-REEP1/6-turboID were treated with a biotin solution and subsequently exposed to 42 μg/mL digitonin solution at 4°C with gentle agitation at 100 r/min for 10 min, following the protocol (Liu and Fagotto 2011). This step effectively removed cytosolic components while preserving membrane-associated proteins.

X

Western blot analysis demonstrated the successful removal of cytosolic proteins: the cytosolic marker GAPDH was absent in the digitonin-treated samples. Meanwhile, components from the nuclear, plasma membrane, cytoskeleton, ER membrane, and mitochondrial membrane fractions were retained, as confirmed by the presence of their respective marker proteins (Figs. 2B and 2C). Since actin is predominantly localized in the cytoplasm and partially interacts with membrane proteins, a small residual amount is expected to remain after digitonin permeabilization (Liu and Fagotto 2011). Notably, the plasma membrane-localized lipid-binding protein Caveolin-1 was detected at elevated levels in the supernatant of the cell lysate from the digitonin-treated group. This is likely due to digitonin’s strong binding affinity for cholesterol, which may reduce the levels of certain proteins in the insoluble fraction following the addition of RIPA buffer.

Taken together, these results confirmed that digitonin treatment effectively permeabilized the cells, selectively isolating membrane-bound, biotinylated proteins. This experiment was performed in triplicates, and the isolated biotinylated proteins were subject to mass spectrometry analysis.

### Data analysis

**Figure 3:**
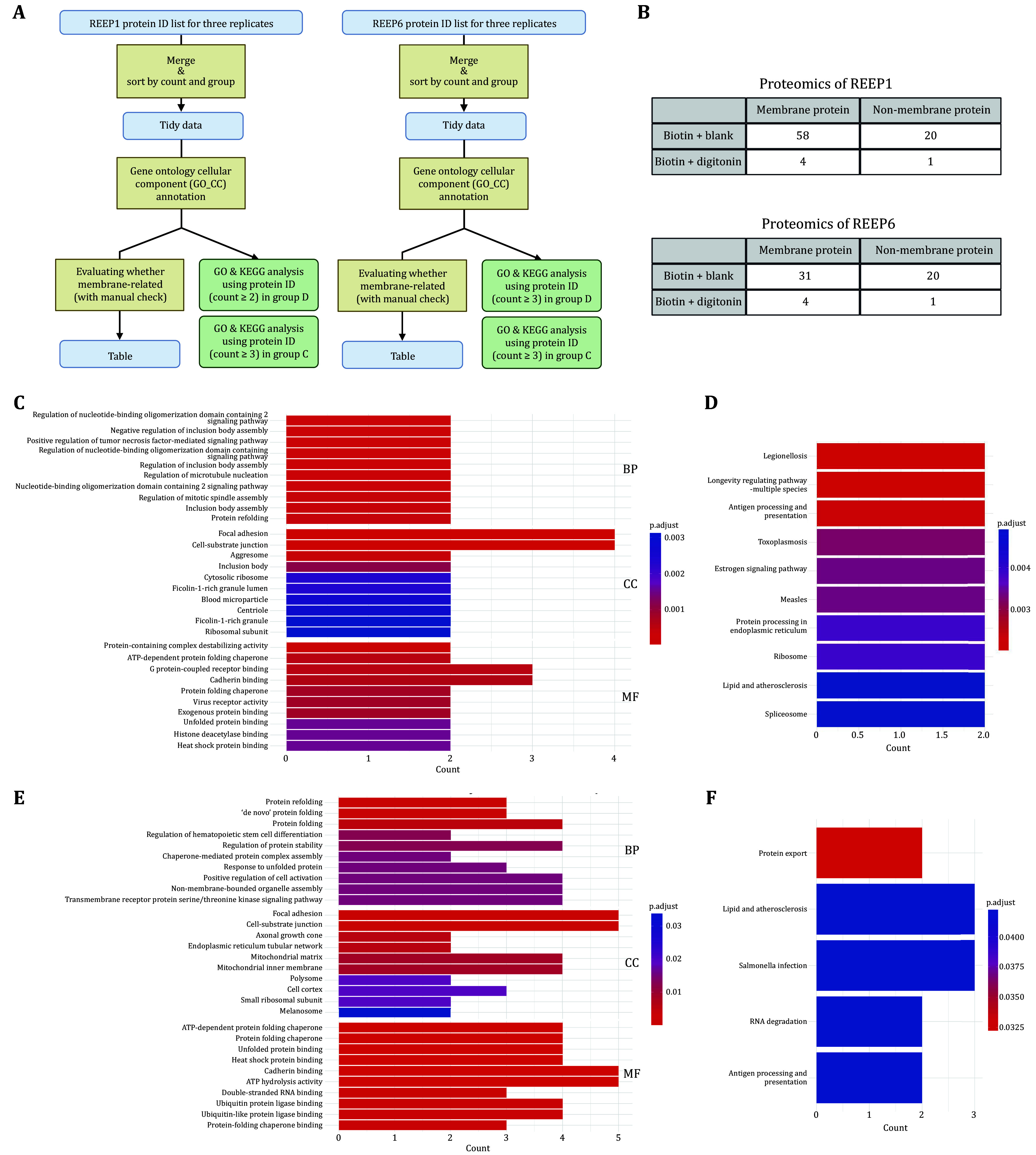
Proteomics data analysis. **A** Workflow illustrating the analysis of mass spectrometric data by using GO and KEGG. Created in BioRender. **B** Comparison of membrane and non-membrane protein counts with or without digitonin treatment. **C**,**E** GO enrichment analysis of REEP1 and REEP6 proteomics. **D**,**F** KEGG pathway analysis of REEP1 and REEP6 proteomics, highlighting their distinct roles in various cellular processes

The raw mass spectrometry data were processed using a protein database search and analyzed using Gene Ontology (GO) and Kyoto Encyclopedia of Genes and Genomes (KEGG) pathway enrichment tools (Fig. 3A). A comparison of membrane versus non-membrane protein counts between digitonin-permeabilized and non-permeabilized samples highlighted the efficacy of digitonin in minimizing cytosolic contamination. Specifically, in the REEP6 interactome, digitonin treatment significantly increased the proportion of membrane proteins from 60.78% (31 out of 51 proteins) in the untreated group (Group C) to 93.33% (14 out of 15 proteins) in the permeabilized group (Group D) (Fig. 3B). This reflects a statistically significant reduction in non-membrane proteins following permeabilization.

Further bioinformatic analysis provided insights into the distinct functional roles of REEP1 and REEP6 in various cellular processes (Figs. 3C**–**3F). REEP1 was closely linked to immune response and cellular structure regulation, with involvement in pathways such as nucleotide-binding oligomerization domain (NOD) signaling, microtubule nucleation, cellular adhesion, extracellular matrix interactions, protein folding, and receptor signaling. KEGG pathway analysis underscored REEP1’s importance in ER protein processing and antigen presentation pathways.

In contrast, REEP6 was predominantly associated with maintaining protein homeostasis, significantly contributing to protein folding and cellular structure stabilization. KEGG analysis further confirmed REEP6’s involvement in protein export, RNA degradation, and immune response pathways, highlighting its regulatory role in key processes that supported proper cellular function.

## DISCUSSION

We have developed a protocol that combines TurboID-based proximity labeling with digitonin permeabilization to map organelle-localized protein interactomes. This approach enabled us to successfully investigate the interaction networks of the ER membrane-shaping proteins REEP1 and REEP6, providing new insights into their roles in membrane dynamics and organization.

The unique structural and functional characteristics of membrane-bound organelles may influence the performance of this technique. For example, mitochondrial membrane proteins are integrated into highly dynamic lipid bilayers, which may affect their interaction dynamics and accessibility. In contrast, lysosomal proteins operate within an acidic, protease-rich environment, potentially influencing their stability and reactivity. Additionally, the intricate architecture of the Golgi apparatus can limit the accessibility of labeling reagents and may hinder the detection of transient or weak interactions. While our method has been successfully applied to ER membrane proteins, further validation is needed to expand its applicability to other organelles. Moreover, the formation of functional membrane contact sites (MCSs) typically involves not only transmembrane proteins embedded in the interacting organelles but also cytosolic effector proteins that are dynamically recruited to these sites. Some of these cytosolic factors may not be captured by this method, which could limit its ability to fully reflect the complexity of MCS components.

Furthermore, while methods such as TurboID offer high spatial resolution and the ability to capture transient interactions, their labeling efficiency can vary depending on the local environment of the target protein, potentially leading to variability in results. Additionally, nonspecific labeling can occur, which may complicate data interpretation (Branon *et al.* 2018; Roux *et al.* 2012). The dynamic nature of membrane protein interactions, influenced by factors such as lipid composition, post-translational modifications, or cellular stress, can also affect the reproducibility and accuracy of results (van Meer *et al.* 2008). To address these limitations, integrating proximity labeling with complementary approaches, such as cryo-electron microscopy, co-immunoprecipitation, or super-resolution imaging, could provide a more comprehensive and accurate characterization of membrane protein interaction networks. This multi-faceted approach would enhance the resolution and depth of our understanding of the complex interactions within cellular membranes.

In conclusion, this protocol offers valuable insights into mapping organelle membrane protein interactomes. With further optimization and validation, it holds substantial potential for widespread application in unraveling protein interactomes across various organelle systems, thereby advancing our understanding of cellular function and inter-organelle communication.

## MATERIALS AND EQUIPMENT

### Biological materials

HEK293T cells were obtained from ATCC (CRL-3216), and U2OS cells were from the Cell Bank, Chinese Academy of Sciences (Shanghai, China). TurboID was amplified from 3× HA-TurboID-NLS (addgene, #107171). pCMV-Myc-REEP1 and pCMV-Myc-REEP6 are generated in the lab. The DNA sequences of Myc-REEP1 or Myc-REEP6 and TurboID were cloned into the lentiviral expression vector (plvx-IRES-Puro) with packaging plasmids (pMD2.G and psPAX2), which were kind gifts from Prof. Du Feng (Guangzhou Medical University, Guangzhou, China).

### Reagents

• Poly-L-Lysine (Sigma, Cat. No. P6282)

• Biotin (Sigma, Cat. No. B4501)

• Digitonin (Sigma, Cat. No. D141)

• Sodium Deoxycholate (Sangon Biotech, Cat. No. A100613)

• Protease Inhibitor Cocktail (Selleck, Cat. No. B14002)

• PMSF (Beyotime, Cat. No. ST506)

• KCl (Sangon Biotech, Cat. No. A100395)

• Na_2_CO_3_ (Sangon Biotech, Cat. No. A500840)

• Urea (Sangon Biotech, Cat. No. A600148)

• NaCl (Hushi, Cat. No. 10019318)

• Tris-HCI (Sangon Biotech, Cat. No. A600194)

• Streptavidin Beads 6FF (Smart Lifesciences, Cat. No. SA021005)

• Formaldehyde (Sigma, Cat. No. P6148)

• Triton X-100 (Diamond, Cat. No. A110694)

• MgCl_2_ (Sangon Biotech, Cat. No. A610328)

• DTT (Beyotime, Cat. No. ST043)

• PEI 25K (Polysciences, Cat. No. 23966-1)

• Opti-MEM (Gibco, Cat. No. 31985070)

• FBS (Vistech, Cat. No. SE100-011)

• Penicillin-streptomycin (Gibco, Cat. No. 15140122)

• Trypsin (Promega, Cat. No. V5280)

• DMEM (Sigma, Cat. No. D6429)

• Puromycin (Sangon Biotech, Cat. No. A610593)

• DAPI (Solarbio, Cat. No. C0060)

• BSA (Sangon Biotech, Cat. No. A600332)

• Rabbit anti-Myc (CST, Cat. No. 2278)

• Mouse anti-GAPDH (Santa Cruz, Cat. No. SC-32233)

• Mouse anti-Tom20 (Santa Cruz, Cat. No. SC-17764)

• Mouse anti-Actin (Santa Cruz, Cat. No. SC-376421)

• Rabbit anti-Sec61β (ABclonal, Cat. No. A15788)

• Rabbit anti-Lamin B1 (CST, Cat. No. 17416)

• Rabbit anti-Caveolin-1 (CST, Cat. No. 3267)

• IRDye 800CW Donkey Anti-Mouse (LI-COR Biosciences, Cat. No. 926-32212)

• IRDye 800CW Donkey Anti-Rabbit (LI-COR Biosciences, Cat. No. 926-32213)

• Donkey Anti-Rabbit Alexa Fluor 555 (Invitrogen, Cat. No. A32794)

• HRP-Conjugated Streptavidin (Invitrogen, Cat. No. N100)

• BeyoECL Moon Kit (Beyotime, Cat. No. P0018FM)

• Phanta Super-Fidelity DNA Polymerase (Vazyme, Cat. No. P501-05)

• Gel DNA Extraction Kit (Vazyme, DC301-01)

• Primary antibody dilution buffer (Beyotime, Cat. No. P0273)

• BCA Protein Quantification Kit (Vazyme, Cat. No. E112-01)

• Fluoromount-G (SouthernBiotech, Cat. No. 0100-01)

• TEAB (Sigma-Aldrich, Cat. No. 18597)

• iTRAQ Reagent-8 plex Multiplex Kit (AB SCIEX, Cat. No. 4381663)

• MMTS (Sigma-Aldrich, Cat. No. 64306)

• TCEP (Sigma-Aldrich, Cat. No. 75259)

• Formic acid (Thermo Scientific Pierce, Cat. No. 28905)

• Acetonitrile (Thermo Scientific, Cat. No. A955-4)

### Equipment

• Rotating culture mixer (Kylin-Bell Lab Instruments, QB-128)

• CO_2_ incubator (Thermo Scientific, BB150)

• Biosafety cabinet (Thermo Scientific, 1300 series A2)

• NanoDrop (Thermo Scientific, 701-058112)

• Decoloring shaker (Kylin-Bell Lab Instruments, TS-2)

• Shaker (Minquan, MQD-S2R)

• Ultralow temperature centrifuge (Thermo Scientific, 21R)

• Centrifuge (Thermo Scientific, PICO 17)

• Metal bath (MIULAB, DKT200-2)

• DNA gel imaging system (SageCreation, ChampGel7000)

• Confocal microscope (Carl Zeiss, 880)

• Western blot imaging system (LI-COR Biosciences, CLx)

• Chemiluminescent Imaging and Analysis System (Sinsage, MiniChemi)

• pH meter (Sartorius, PB-10)

• Magnetic Separation Rack (Selleck, Cat. No. B23803)

• Glass coverslips (WHB, Cat. No. WHB-12-CS)

• PVDF (Millipore, Cat. No. IEVH85R)

• Thermal Cycler (Bio-Rad, T100)

• Mini Trans-Blot Cell (Bio-Rad, 1703935)

• Mini-PROTEAN Tetra Cell (Bio-Rad, 1658001)

• TripleTOF 5600+ System (AB SCIEX)

• NanoLC.2D (Eksigent Technologies, 3C18-CL)

• 10 K filter (Vivacon)

• Durashell C18 column (Agela, Cat. No. DC952505)

• nanoLC column (AB SCIEX, Cat. No. 805-00120)

• High-performance liquid chromatography (HPLC) system (SHIMADZU)

• SpeedVac (Labconco)

• NanoLC.2D system (Eksigent Technologies)

• ProteinPilot Software (AB SCIEX, version 4.5)

### Reagent setup

(1) Complete medium (10% FBS, 20 U/mL Penicillin-streptomycin, DMEM). To prepare 500 mL complete medium, mix 50 mL FBS and 1 mL of 2000 U/mL Penicillin-streptomycin in 500 mL DMEM. Store at 4°C.

(2) 100 mmol/L Biotin: Dissolve 100 mg biotin in 4.093 mL DMSO to a final concentration of 100 mmol/L. Store at −20°C.

(3) 10× NEH buffer (1500 mmol/L NaCl, 2 mmol/L EDTA, 200 mmol/L HEPES-NaOH, pH 7.4). To prepare 50 mL of 10× NEH buffer, mix 4.385 g of NaCl, 20 μL of 0.5 mol/L EDTA, 5 mL of 1 mol/L HEPES-NaOH, and bring the solution to a final volume of 50 mL with bi-distilled water and adjust the pH to 7.4 using 1 mol/L NaOH. Store at 4°C.

(4) Digitonin purification. Dissolve 50 mg of commercial digitonin in 1.25 mL of absolute ethanol at 75°C. Chill the solution at 0°C (ice water) for 20 min and then separate by centrifugation at 4°C. This procedure is repeated twice, resulting in approximately 60% recovery obtained after vacuum drying. This grade of digitonin can be readily dispersed in bi-distilled water (42 mg/mL), yielding a clear apparent solution that will become turbid after 1**–**2 h of standing, thus, only fresh solutions should be used.

(5) Digitonin solution (42 μg/mL digitonin, 2 mmol/L DTT, 2 mmol/L MgCl_2_ in 1× NEH buffer). To prepare 50 mL of digitonin solution, mix 50 μL of 42 mg/mL digitonin, 100 μL of 1 mol/L DTT, and 100 μL of 1 mol/L MgCl_2_ and bring the final volume to 50 mL with 1× NEH buffer. This buffer needs to be prepared freshly on the day of the experiment.

(6) RIPA Lysis Buffer (50 mmol/L Tris-HCl, 150 mmol/L NaCl, 0.1% SDS, 0.5% sodium deoxycholate, 1% Triton X**-**100, 1× protease inhibitor cocktail, 1 mmol/L PMSF). To prepare 50 mL of RIPA lysis buffer, mix 2.5 mL of 1 mol/L Tris-HCl (pH 8.0), 1.5 mL of 5 mol/L NaCl, 500 μL of 10% SDS, 250 μL of 10% sodium deoxycholate, 2.5 mL of 20% Triton X-100 and 500 μL 100x protease inhibitor cocktail and 50 μL of 1 mol/L PMSF, bring the volume to 50 mL with bi-distilled water. This buffer needs to be prepared freshly on the day of the experiment.

(7) 1 mol/L KCl: Dissolve 3.73 g potassium chloride (KCl) in 50 mL distilled water to a final concentration of 1 mol/L. Store at room temperature.

(8) 0.1 mol/L Na₂CO₃: Dissolve 0.53 g sodium carbonate (Na₂CO₃) in 50 mL distilled water to a final concentration of 0.1 mol/L. This solution should be prepared fresh before use.

(9) 1 mol/L DTT Stock: Dissolve 3.09 g dithiothreitol (DTT) in 20 mL distilled water. This solution can be stored at −20°C for several months.

(10) 1 mol/L MgCl₂: Dissolve 1.02 g of magnesium chloride hexahydrate (MgCl₂-6H_2_O) in 5 mL of distilled water. Sterilize the solution and store it at room temperature.

(11) 2 mol/L Urea solution (2 mol/L Urea in 10 mmol/L Tris-HCl, pH 8.0). To prepare 50 mL of 2 mol/L urea in 10 mmol/L Tris-HCl, dissolve 6 g of urea in 40 mL bi-distilled water and add 500 μL of 1 mol/L Tris-HCl, bring the solution to a final volume of 50 mL with bi-distilled water and adjust the pH to 8.0, storing at 4°C.

(12) 1 mol/L HEPES-NaOH Solution. To prepare 50 mL of 1 mol/L HEPES-NaOH solution, dissolve 111.915 g of HEPES in 40 mL of bi-distilled water, bring the solution to a final volume of 50 mL with bi-distilled water, and adjust the pH to 7.4 using NaOH. Store the solution at 4 °C.

## Conflict of interest

Yang Sun, Lan Yang, Jingzi Zhang, Pu Tian, Shue Chen, Lei Fang and Zhi Hong declare that they have no conflict of interest.
